# Perceptions of risk and coping strategies during the COVID-19 pandemic among women and older adults

**DOI:** 10.1371/journal.pone.0301009

**Published:** 2024-04-17

**Authors:** Guek Nee Ke, Alan Gow, Rachel Mei Ming Wong, Shahirah Raman, Zulaikha Mohammad, Nicole De-Lima, Rozainee Khairudin, Wee Yeap Lau, Khalil Anwar Kamal, Shen Chiang Lee, Dasha Grajfoner

**Affiliations:** 1 Department of Psychology, School of Social Sciences, Heriot-Watt University, Putrajaya, Malaysia; 2 Centre for Applied Behavioural Sciences, Heriot-Watt University, Putrajaya, Malaysia; 3 Department of Psychology, School of Social Sciences, Heriot-Watt University, Edinburgh, United Kingdom; 4 National University Malaysia (UKM), Bangi, Malaysia; 5 Faculty of Economics and Administration, Department of Applied Statistics, University Malaya, Kuala Lumpur, Malaysia; 6 Malaysian Institute of Economic Research (MIER), Kuala Lumpur, Malaysia; 7 DOBA Business School, Maribor, Slovenia; Dow University of Health Sciences, PAKISTAN

## Abstract

The world’s health, economic, and social systems have been adversely impacted by the COVID-19 pandemic. With lockdown measures being a common response strategy in most countries, many individuals were faced with financial and mental health challenges. The current study explored the effect of the COVID-19 pandemic on the psychological well-being, perception of risk factors and coping strategies of two vulnerable groups in Malaysia, namely women and older adults from low-income households (USD592). A purposive sample of 30 women and 30 older adults was interviewed via telephone during Malaysia’s Movement Control Order (MCO) regarding the challenges they faced throughout the pandemic. Thematic analysis was subsequently conducted to identify key themes. The themes identified from the thematic analysis indicated a degree of overlap between both groups. For women, seven themes emerged: 1) Psychological challenges due to COVID-19 pandemic, 2) Family violence, 3) Finance and employment related stress and anxiety, 4) Women’s inequality and prejudice, 5) Coping strategies, 6) Professional support, and 7) Women’s empowerment. Similarly, there were six themes for the older adults: 1) Adverse emotional experiences from COVID-19, 2) Threats to health security, 3) Loss of social connections, 4) Government aid to improve older adults’ psychological well-being, 5) Psychological support from family members and pets, and 6) Self-reliance, religion, and spirituality. The findings provide valuable information on the specific burdens faced by these groups, and support psychological interventions and mitigations that would be appropriate to improve well-being during the recovery phase.

## Introduction

The spread of Coronavirus 19 (COVID-19) impacted health, social, and economic systems around the world. Countries urgently implemented movement restriction orders (i.e., lockdowns), which were successful at breaking the chain of transmission. However, these interventions were not without adverse effects, as seen in increased levels of depression, anxiety, and stress [1–4]. Research has mostly focused on Western countries, with regions including Southeast Asia understudied [5]. Research that has been conducted in Southeast Aisa has predominantly considered the environmental, financial, and economic repercussions of COVID-19 rather than the well-being of citizens [6–8].

In Malaysia, the implementation of the Movement Control Order (MCO) in March 2020 successfully curbed the spread of COVID-19 [9–11], but contributed to other adverse economic and psychological outcomes [3,4,12–14]. For example, research has considered the economic impact of COVID-19 in Malaysia [15–17]. In response to those impacts, the Malaysian government directed resources to alleviate economic hardships through stimulus programs (e.g., PRIHATIN, Penjana). When studies considered psychological well-being, those were more likely to investigate student well-being [18,19], frontline health workers [20–22], and have a cross-sectional methodology [23–25]. While those studies described the impact COVID-19 had, there was less consideration given to identifying potential avenues of interventions. There is, therefore, a significant gap in research: the lack of qualitative data on the experiences of COVID-19 faced by vulnerable Malaysians (specifically those from low-income households), and the coping strategies they may have employed to cope with those challenges.

The current study addressed this by conducting qualitative interviews with Malaysian women (N = 30) and older adults (N = 30) from low-income households. The findings provide information on the specific burdens faced by these groups, and suggestions for psychological interventions that could be appropriate to improve well-being.

### Vulnerable groups in Malaysia

The Malaysian government recognises women, older people, people with disabilities, the poor, youth, refugees, and immigrants as vulnerable groups, in accordance with the United Nations Electronic Government Development Index Survey (UNEG-DI) [26]. Unlike the general population, these vulnerable groups have limited access to resources (e.g., healthcare, employment), and face increased social injustices (e.g., domestic abuse, disability), resulting in a high prevalence of poverty and social exclusion [27,28]. There are also prevailing socioeconomic inequalities in these vulnerable groups versus the general population, which were exacerbated due to COVID-19. For example, the number of low-income (B40) households, classified as those earning less than RM2,500 (approximately USD592) a month, increased by 12.5% [12,29]. Although the World Bank’s High Frequency (HiFy) Household Monitoring Survey reported that COVID-19 negatively impacted all Malaysian households, the most adversely affected were those which were disadvantaged before the pandemic; limited resources became scarcer still due to the lack of support and income [30]. Given the importance of disadvantage in predicting outcomes from COVID-19, the current study focusses on the low-income (i.e., B40) population, and specifically women and older adults as women are disproportionately disadvantaged due to social structures in Malaysia, and older adults were more socially isolated due to their higher physical risk of infection.

#### Women in Malaysia during MCO

The adverse physical and mental impact of COVID-19 on women has been well-documented, from increased domestic violence to loss of employment and income [31,32]. For Malaysian women, pre-existing inequalities were compounded by a reduction in opportunities in the labour market [33,34]. The Statistics Department of Malaysia reported that women made up nearly 70% of unemployed individuals in 2020; the lack of labour force participation was attributed to domestic and caregiving responsibilities [35–38]. The lockdown also increased the likelihood of gender-based domestic violence, with the Woman’s Aid Organisation (WOA) reporting a 44% increase in the number of calls received as women were unable to leave their abusers due to limited access to resources [39]. This increase is not atypical, comparable to the 10–50% increase in domestic violence helpline calls in other countries [40]. Financial stress and economic insecurity from the pandemic have been identified as key contributing factors to the increase in domestic violence in Malaysia [41,42].

Gender-based inequalities can be traced back to Malaysia’s predominantly patriarchal culture [43], which was exemplified during the MCO when the government mandated that only head of households–who are typically male–were allowed to leave the home to shop for the family’s essential needs; women were unable to do so for themselves or their family [44]. Gynocentric policies such as these during times of crisis further reinforced gender stereotypes and contributed to increased gender inequality in Malaysia [45].

Due to these challenging circumstances, a higher proportion of women than men experienced negative emotions, and severe or extremely severe signs of depression, anxiety, and stress throughout the MCO [46,47]. Despite these trends, limited qualitative research has been conducted to consider gender-specific responses that would support the well-being of women [34,48].

#### Older adults in Malaysia during MCO

Older adults were also specifically vulnerable to the effects of COVID-19 related restrictions, as the decreased social contact and increased isolation during MCO accelerated changes in their mental and physical health [49–52]. Older adults are defined as those aged 60 years or older [53], and an ageing society as a nation with a population where more than 7% are considered older adults [54]. Malaysia’s population surpassed this criterion in 2020, making the country an ageing nation, and it is projected to increase to 15% by 2030 [55]. The increase in the proportion and therefore absolute number of older people in the population supports the specific consideration of their evolving needs and requirements, particularly the interaction between social, economic, political, and environmental variables with health and function [56–58]. As older adults were considered a high-risk group during the COVID-19 pandemic, many of the MCO’s standard operating procedures (SOP) were designed to reduce exposure by keeping them physically distanced from family and friends [9]. Although these guidelines protected older adults physically, it resulted in unintended health risks, as many became physically and socially inactive following self-isolation [51,52,59]. Social isolation has been associated with both chronic physical conditions, such as diabetes and cardiovascular disease, and poorer mental health [60–62]. Recent studies indicate that older adults in Asian countries experienced impairments in cognitive function [51], and depressed mood and frailty [52] during the COVID-19 pandemic with social isolation often suggested as a key contributor.

Despite its necessity, the enforcement of social distancing and lockdown measures reduced social connections, with consequences for mental health [63]. Based on relational-cultural theory [64], interdependent experiences of connection, based on mutual empathy and mutual empowerment, enhance resilience and contribute to personal growth [65–68]. In isolation, individuals tend to repeat old patterns or thoughts and often feel disempowered [64]. These feelings of disempowerment are particularly detrimental for older adults, whose sense of self and worth and well-being are built upon their connections with others [69].

## Conceptual model

### Women and older adults

Prior to the data collection and analysis, the researchers proposed two theoretical frameworks: the socio-ecological model [70] and the emotional-transactional model [71]. Given the severity of COVID-19 and the impact on multiple aspects of daily life, considering both the emotional and non-emotional implications of lockdown were relevant.

### Socio-ecological model

The complex interplay between Malaysian women and the patriarchal society suggests that a multi-level explanation would be appropriate to understand factors that could alleviate the challenges faced. The socio-ecological model, which has been utilized amongst vulnerable groups [72], provides a concise framework that arranges possible barriers to assistance for vulnerable groups in Malaysia.

The theory-based model conceptualises the interaction between an individual and their environment by proposing four nested levels–individual, relationship, community, and society–and respective risk and protective factors [73]. The model consists of four concentric circles, beginning with the innermost circle (individual) that represents an individual’s biological or personal history. The subsequent circle (relationship) embodies an individual’s relationship with those they consider close, such as family or friends. The third level (community) involves social structures and any formal or informal institutions which the individual has a relationship with. The outermost circle (society) typifies the social and cultural environment, which includes cultural norms, laws, and policies. According to this approach, the challenges faced by women and the severity of those will be dependent on the interaction between these four factors.

**Fig 1:**
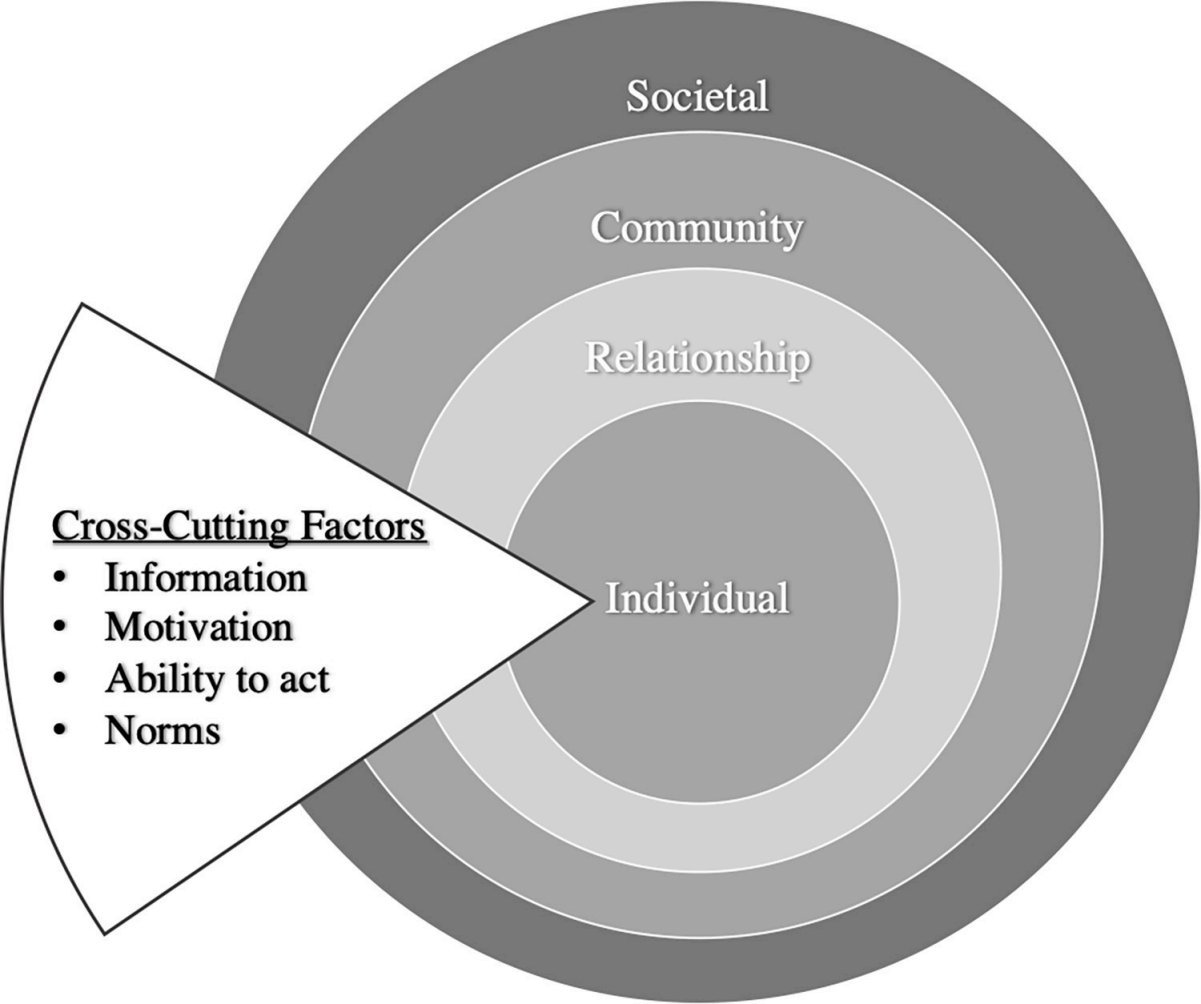
Socio-ecological model for change.

An updated model (Socio-Ecological Model for Change) [74] introduces an additional component into: the cross-cutting factors (Fig 1). These cross-cutting factors essentially represent opportunities for change and mitigation throughout the structure of the rings, identified as: information, motivation, ability to act, and norms.

In this study, the socio-ecological model for change would organize the negative experiences of the MCO into the concentric levels, and the participants’ coping mechanisms into the cross-cutting factors.

### Emotional-transactional model

**Fig 2:**
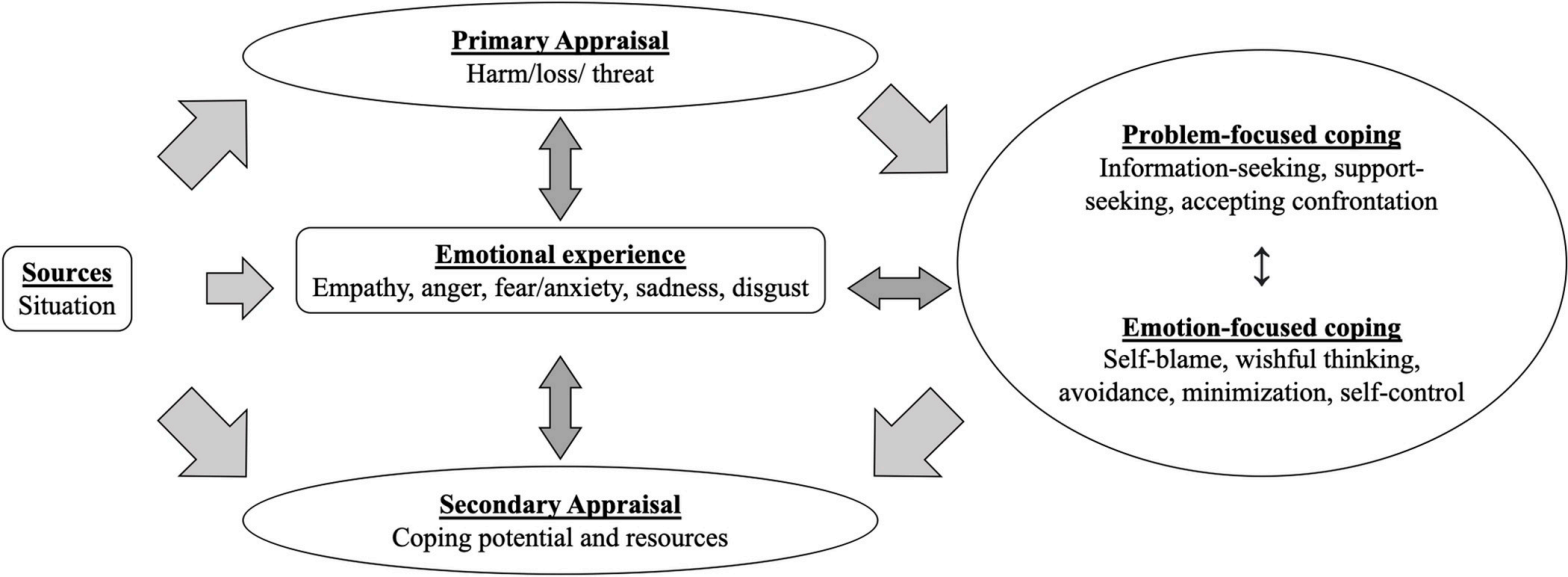
Emotional-transactional model.

The emotional-transactional model integrates the transactional theory of stress [75] with the cognitive-motivational-relational theory of emotion [76] (Fig 2). In the primary appraisal phase, a situation is evaluated as being a threat, harm/loss, or a challenge, whereas secondary appraisal involves identifying potential coping resources. This theory differs from that of the original transactional theory of stress in that the coping process occurs after the appraisal process and emotional experience. Previously, emotion was proposed as the outcome of cognition, and remained neglected despite its potential importance in the appraisal process [77–79]. However, the emotional-transactional model expands on Lazarus’ previous work and includes the four basic ‘negative’ universal emotions: fear/anxiety, disgust, anger, and sadness [80]. This provides a more comprehensive view of the appraisal process that an individual engages in when confronted with a stressful situation.

### Current study

Women and older adults were disproportionately vulnerable during the COVID-19 pandemic. In Malaysia, women were culturally and socially exposed to multiple inequalities due to increased domestic responsibilities and economic and marital stressors, whilst the growing ageing population had higher health risks coupled with more limited access to social support. These negative impacts are compounded when considering low-income (i.e., B40) households. The current study therefore explored risk factors for the negative impact of the MCO within these two groups. Semi-structured interviews were conducted to allow more in-depth accounts of the experiences faced by women and older adults in low-income (i.e., B40) households during the MCO, to provide suggestions for possible coping mechanisms and develop future policy recommendations that would protect their interest and needs.

## Materials and methods

### Ethical considerations

Prior to data collection, ethical approval was provided by the School of Social Sciences’ Ethics Committee at Heriot-Watt University (2020-0461-1466).

### Research design

The study used an exploratory qualitative research design to gather information regarding the psychosocial experiences of women and older adults during COVID-19. Interpretative phenomenological analysis (IPA) [81] was conducted, with the two-stage interpretation process (also known as double hermeneutic) allowing participants to first make sense of their personal experiences prior to researchers making sense of participants’ descriptions. A semi-structed interview was conducted, with prompts provided to encourage participants to share more information (S1 Appendix). This method was selected as it produces rich descriptions of personal experiences that could be enhanced by further questioning by the researchers [82].

### Participants

Participants were required to be above the age of 18 for women, and 65 or older for older adults. Individuals who self-reported any mental health challenges were not eligible to participate. Both sets of participants were also asked to self-reported how their living experience was affected by the COVID-19 lockdown, but they were not required to report any physical health challenges.

The sample consisted of Malaysian citizens who were from low-income households (i.e., B40, household that earn less than RM2,500 (approximately USD 592) per month). Participants in the women group (N = 30) were ageds 19 to 59 (M = 30.28, SD = 11.25), and consisted of Malays (N = 10), Indians (N = 10), Chinese, (N = 6), and Bornean (N = 4). Participants in the older adult group (N = 30, N_female_ = 15, N_male_ = 15) were aged 60 to 79 (M = 67, SD = 5.11), with an equal representation of Malays (N = 10), Indians (N = 10) and Chinese, (N = 10). All participants reported some form of financial loss throughout MCO, be it through the loss of employment or reduction in income and/or in savings. Approximately 75% (N = 45) of participants received some form of government aid (e.g., Prihatin, State Government Aid).

### Data collection

Due to the MCO, recruitment was conducted remotely, with posters distributed digitally to several non-governmental organisations (NGOs) (e.g., Malaysian Institute of Economic Research, MyKasih, Ministry of National Unity & Integration (PERPADUAN)) and old folks homes. Interested participants were directed to contact the research team, who provided further information and arranged an interview for those who agreed to participate.

To adhere to COVID-19 safety restrictions, data collection was conducted by telephone. Prior to the interview, participants were emailed a participant information sheet and consent form. These documents informed participants of their rights and the procedure for the audio-recorded interview which would last approximately 60 minutes. If participants agreed to proceed with the interview, they were required to digitally sign and return the written informed consent form to the researcher via email. Interviews took place between 21^st^ June and 15^th^ July 2020, while Malaysia was still under MCO, and conducted by two research assistants.

### Data analysis

All interviews were transcribed verbatim, anonymised, and checked for accuracy by the principal investigators prior to being analysed. The information was coded, analysed for patterns, and developed into themes (and sub-themes) by the research assistants using NVIVO (version 12). The results were further assessed for correctness by the fuller research team, with themes and sub-themes rigorously reviewed and amended where necessary, until they adequately represented the dataset.

## Results

The thematic analysis determined that for women, the socio-ecological model was better suited to their experiences, whilst the emotional-transactional model was a better fit for older adults. The demographic characteristics of participants are summarized in Table 1.

**Table 1:** Demographic characteristics of participants.

Demographic	Women		Older adults	
	N	%	N	%
**Gender** Male Female	- 30	- 100	15 15	50 50
**Marital status** Married Single Others	5 22 3	16.7 73.3 10.0	19 3 8	63.3 10.0 26.7
**Education** No formal education/up to college University	16 14	53.3 46.7	26 4	86.7 13.3
**Ethnicity** Malay Chinese Indian Others	10 6 10 4	33.3 20.0 33.3 13.3	10 10 10 -	33.3 33.3 33.3 -
**Locality** Rural Urban	4 26	13.3 86.7	7 23	23.3 76.7
**Reduction of income during COVID-19** 0–50% 51–75% >75%	13 4 13	43.3 13.3 43.3	15 5 10	50.0 16.7 33.3

*Note*: For Education, University refers to participants who had an undergraduate or postgraduate level qualification.

### Women

**Fig 3:**
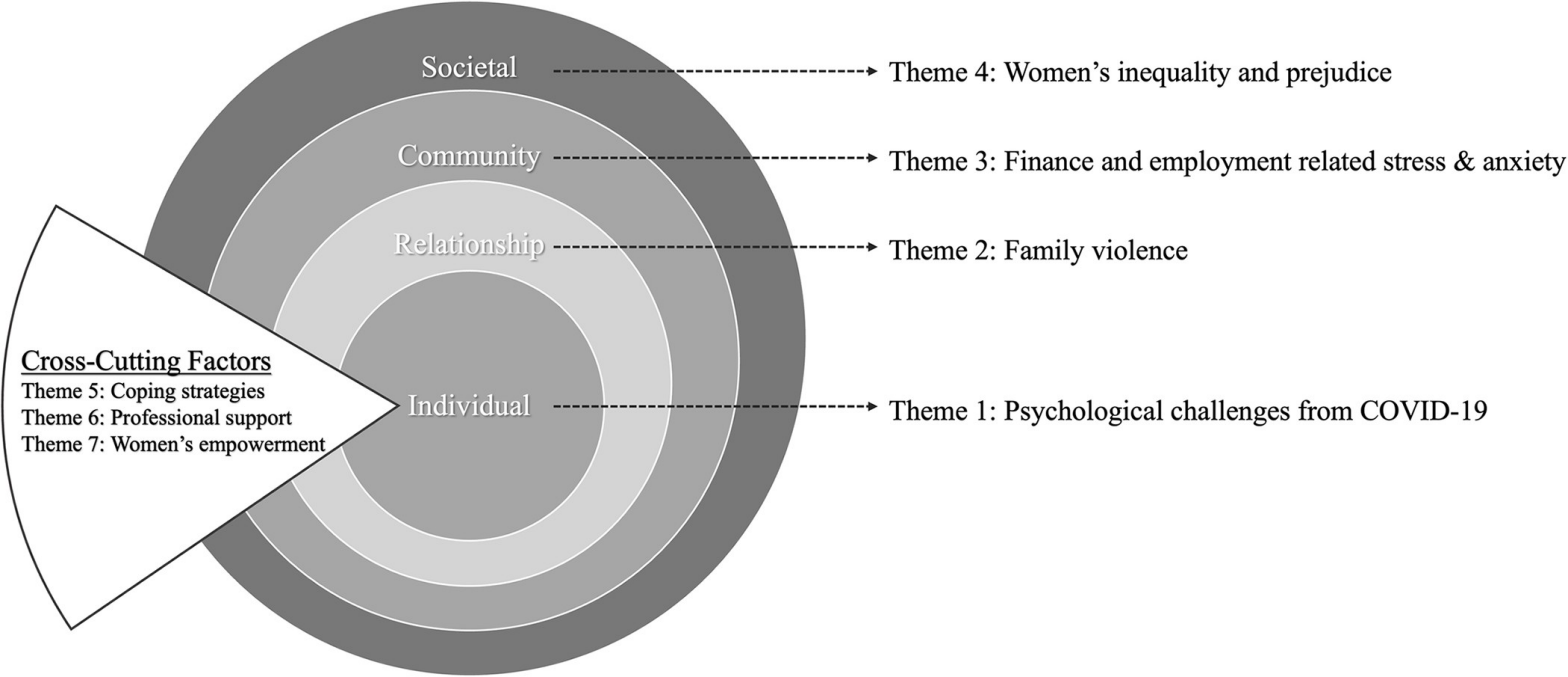
A socio-ecological model of the impact of COVID-19 on vulnerable Malaysian women.

The thematic analysis revealed seven themes that were: (1) Psychological challenges due to COVID-19 pandemic, (2) Family violence, (3) Finance and employment related stress and anxiety, (4) Women’s inequality and prejudice, (5) Coping strategies, (6) Professional support, and (7) Women’s empowerment. The seven themes and their sub-themes are discussed in detail below, with verbatim quotes from the interviews to illustrate each point. The themes are organised and presented according to the individual, relationship, community, and societal levels of the ecological framework, and the cross-cutting factors of the socio-ecological model framework (in Fig 3, the themes are overlaid on the socio-ecological model). Extracts and quotes from participants are italicised, and participants numbers are presented with the prefix P (e.g., P06 refers to Participant 6).

#### Individual level

*Psychological challenges due to COVID-19 (Theme 1)*. At an individual level, participants reported anxiety when coming in close contact with others for fear of contracting COVID-19 themselves, and infecting others in their household. They were particularly worried about spreading the infection to those at higher risk of becoming severely ill with COVID-19, including older relatives and contacts, children, and people with chronic illness.

“*Some of the people close to me are vulnerable*, *and I could be just fine but still be carrying the virus*. *So*, *that’s pretty scary*.*”* (P062)

Notably, participants displayed awareness of the importance of adhering to the standard operating procedures set by the authorities and took precautions when in public.

“*When the MCO started*, *I was prepared to follow all the guidelines set by the authorities*, *like frequently washing hands*, *wearing face masks and all that*. *I felt like these are important guidelines to follow*.*”* (P085)

Adjusting to changes in lifestyle due to movement restriction measures was a common cause for upset among participants. They expressed “feeling down”, “depressed” and “trapped” by being confined at home. Feelings of loneliness and isolation were also evident because of reduced social interactions with people outside the home.

“*I think right at the forefront is the sense of loneliness and isolation [from] not having any kind of personal connection with people I usually meet and spend time with*.*”* (P070)

In addition, participants reported experiencing a lack of motivation and interest in engaging in their usual activities. Some even cited a general feeling of hopelessness and a loss of meaning in life.

“*[My friend] feels like they’re being locked down and life [has] no meaning… she just doesn’t have that energy it feels to do anything*.*”* (P064)

#### Relationship level

*Family violence (Theme 2)*. At this level, the family posed a significant risk to participants’ well-being, as increasing family tensions were prevalent due to spending more time together at home. This was especially true in intergenerational households, as well as from financial stress. Several participants reported physical violence at the hands of their parents.

“*During the pandemic*, *we are forced to stay with each other*. *And there [has been] a lot of fights that even resulted in physical violence*. *[…] It becomes violent*, *and our youngest in the family is a baby*. *It’s a two year old baby*. *And we’re very fearful that [our mother] will let out her anger on that baby*.*”* (P077)

Due to the movement restriction measures, participants were unable to seek refuge away from family, as they would have before the pandemic.

“*When the MCO started*, *I had to go home from my university*. *Actually*, *for my mental health*, *it’s better for me to be in university because I actually have a lot of problems [with] my household*. *[…] Until now*, *I’m still hoping when for the university to open for me to go back*.*”* (P075)

**“***Before the MCO*, *at least*, *myself or my siblings could escape the household violence by staying over at our friend’s house*, *like we seek shelter there*, *we at least have some sort of relief there*. *But during the MCO*, *[…] we cannot go places so we stay home and we have to endure whatever violence whether mentally or physically in this house*. *There’s no escape*.*”* (P077)

#### Community-level

*Finance and employment related stress and anxiety (Theme 3)*. Participants indicated that at a community level, financial instability was a major stressor in their lives. They expressed significant distress due to financial hardships; with many of them experiencing loss of income due to retrenchment, problems in finding work, and changes in their job situation.

“*I had secured a few jobs to sustain myself until the end of the year*, *and when all of that fell through*, *it got me anxious*. *I really didn’t know what to do*. *I had to think of paying rent*, *getting food*, *paying off the car and all that*.*”* (P066)

Among those employed during the MCO, some described feeling frustrated with working from home and in front of a webcam, which they believed to be a surveillance tactic by employers.

“*I work four days a week in front of a webcam*, *so it takes a huge mental toll because for the whole eight hours you are filled with anxiety and it’s extremely stressful to work that way*.*”* (P074)

Furthermore, a subset of participants reported feeling overwhelmed from an increased burden of unpaid care work, which included household chores, caring for family, and helping their young children with online learning.

“*My workload has increased*. *I’m not able to try doing part-time work or anything because my work full-time is really at home because the children do online studies*, *my husband [is] also in the house and being online*. *So there was hardly time for myself*.*”* (P088)

#### Societal level

*Women’s inequality and prejudice (Theme 4)*. At the beginning of the MCO, the Ministry of Women, Family and Community Development of Malaysia released a set of recommendations for married women on how to manage their households (Law, 2020). The Government encouraged women to wear make-up at home and avoid ‘nagging’ their husbands to help with the household chores, and instead attempt to inject humour by speaking to their husbands while emulating the child-like voice of a popular Japanese children’s cartoon character, Doraemon. Several participants had mentioned feeling degraded and insulted by these recommendations.

“*I think those [recommendations] were very misleading*. *I didn’t feel protected at all [by them]*. *In fact*, *I felt very insulted as a woman and I felt very degraded*.*”* (P077)

*“The women’s ministry was very degrading*. *It was very shameful*. *It’s hard [to believe] a post like that would [be approved] that easily*.*”* (P084)

Participants frequently expressed feeling judged by society and pressured to conform to traditional gender roles and stereotypes, including having to sacrifice their needs to care for others, often being branded as less capable than their male counterparts, subservient, as well as overly dramatic. Participants took the opportunity to highlight the need to take women’s concerns seriously, as well as the importance of women empowerment.

“*I’ve noticed that when a woman says that she’s going [struggling with] mental illness*, *people are easy to dismiss it as a women issue*.*”* (Participant 80)

*“Women need empowerment to face the difficulties in their lives*. *[…] Women face a lot of burdens and we often shut ourselves up*, *and if we don’t*, *we receive backlash*.*”* (P085)

*“I think maybe encourage women to speak up*. *Because I know our society tends to be judgmental towards women*, *especially ones who are vocal*. *And some people might have some difficulties to understand and accept what women are saying*. *I’d like more empowerment for women*.*”* (P066)

#### Cross-cutting factors

*Coping strategies (Theme 5)*. Self-coping strategies. When asked how they coped during MCO, participants emphasised the importance of filling their time with activities. Such activities included watching videos and television, as well as engaging in physical activity and a variety of hobbies. Some women highlighted that religious or spiritual practices helped alleviate their distress.

“*I pray for patience and strength*, *more resilience [to overcome] all the mental challenges that we have to go through*.*”* (P087)

Addressing unhelpful thought patterns, meditation and breathing exercises were also cited as coping strategies.

“*With meditation*, *you get to empty your mind and focus on what’s really troubling you*. *I don’t know how other people do it*, *but that’s what I do*. *I try to focus on solutions*, *instead of the problems*.*”* (P066)

Family, friends, and community support. Participants also revealed that they relied on family, friends and/or community for emotional support during the MCO. They found that opening up and sharing their hardship with people that they trusted or were comfortable with improved their psychological well-being.

“*Honestly just my close friends*, *if anything I would get in contact with them and tell them how I feel and what happened*.*”* (P066)

Furthermore, participants found that joining an online support group helped relieve some of their distress.

“*I came across this website [linking to] a worldwide support system where you can talk to an anonymous person about what you’re feeling*. *You can find someone who’s experiencing the same thing as you*. *And you have the advantage of being anonymous*, *so you don’t have to worry about being looked at in a different way or anything like that*. *I’ve found that this particular website really helped me*.*”* (P080)

*Professional support (Theme 6)*. Financial and career support. To assist with financial and employment issues, participants proposed that financial aid should be given to retrenched workers and those struggling with financial insecurity.

“*[*…*] for financial support*, *it would be in the form of cash aids to help people who struggle to get by on certain financial thresholds*.*”* (P072)

Initiatives to create more job opportunities as well as to support local businesses were also suggested.

“*I want more ways to support local businesses and B40 workers in rural areas and small towns*. *Because some people might be in a more stable position*, *but these folks are just starting to find their footing*.*”* (P068)

Many participants also expressed interest in attending skill-building workshops to improve their employability. Some skills of interest included business, freelancing, communication skills, marketing, advertising, and technological skills, as well as language classes.

Stigma of psychological support and services. Most participants were aware of at least one psychological service available to them. These included mental health hotlines, government or private mental health services, and non-governmental organizations (NGOs). Local suicide hotlines, befrienders, and NGOs including Women’s Aid Organisation (WAO) and the Malaysian Mental Health Association (MMHA) were the most reported among participants. However, only a small number of participants revealed that they had sought psychological support. Some participants disclosed that they were reluctant to seek psychological support despite ongoing mental health issues due to previous negative experiences.

“*Because it hasn’t really been a positive experience for me*. *Even if there are avenues—I don’t want to take them*.*”* (P076)

To address mental health issues, participants highlighted the need to first destigmatize mental illness nationwide and boost mental health education. They further stressed that stigma was a prominent barrier in seeking psychological help.

“*I think after COVID-19*, *people are going to need a lot of mental health help*, *but I don’t know if they are going to be willing to seek mental health since it’s so stigmatized*.*”* (P078)

“*I think that many people are still not aware of [mental illness]*. *People usually think [people with mental health issues] are ‘gila’ [crazy]*. *[…] I think people must be educated*.*”* (P067)

Increase public mental health support and in the workplace. One participant suggested that more mental health programmes specific for women should be made available and that they should be locally relevant.

“*[There should be] workshops highlighting women’s mental health*. *We have an entirely different biological system that is very much affected by our hormonal balance*. *[This is] not made aware to the everyday woman*, *like me*.*”* (P070)

Other participants opined that addressing mental health issues would be more effective when done through one’s social circle (e.g., family, friends, or colleagues) rather than larger bodies.

“*You can actually be the first point of contact*, *helping someone realise that they are going through something*.*”* (P089)

Furthermore, participants also recommended that more mental health services should be initiated as they believed existing services tended to be costly, inaccessible, ineffective or generally difficult to navigate.

“*I think it would be helpful if we could have more counsellors or therapists made available in more places*. *I have [previously] considered getting psychological help*, *but it’s almost impossible*. *If you go to a private therapist*, *it’s expensive*. *If you go to the government one*, *it’s not easily accessible*. *So*, *I think I’d like to see more accessible mental health plans for everyone*.*”* (P074)

Increasing support for mental health in the workplace was also a common suggestion.

“*I just feel like when it comes to employers in Malaysia*, *they are not very understanding when it comes to mental health*. *It would be great if we could have workshops or seminars on how to deal with employees’ mental health*.*”* (P074)

*Women’s empowerment (Theme 7)*. To help women become more empowered, participants proposed launching a platform bringing together a community of women, where they could express their concerns safely and find support.

“*For women*, *especially those who don’t have their own stable income*, *they would need support and empowerment to improve themselves*. *There should be programs for that*.*”* (P073)

“*It would be nice if there was a channel for women to voice out their experiences*, *because right now everyone is going through their own struggle*.*”* (P062)

“*Having more accessible groups or facilities for women to express their concerns*. *[…] If they are facing some sort of emotional issues regarding certain matters*, *there should be a group—a safe group—that could be a channel for these women to express what has been going on*.*”* (P072)

Additionally, several participants also highlighted the need to address violence against women, including domestic violence, sexual harassment and online gender-based violence, and further implored that appropriate assistance be given to those affected by these issues.

“*The most important issue—I think it would be the well-being of the people who have suffered domestic violence*. *[Emphasis] definitely needs to be put on [providing] resources and aid [for these women]*.*”* (P083)

### Older adults

**Fig 4:**
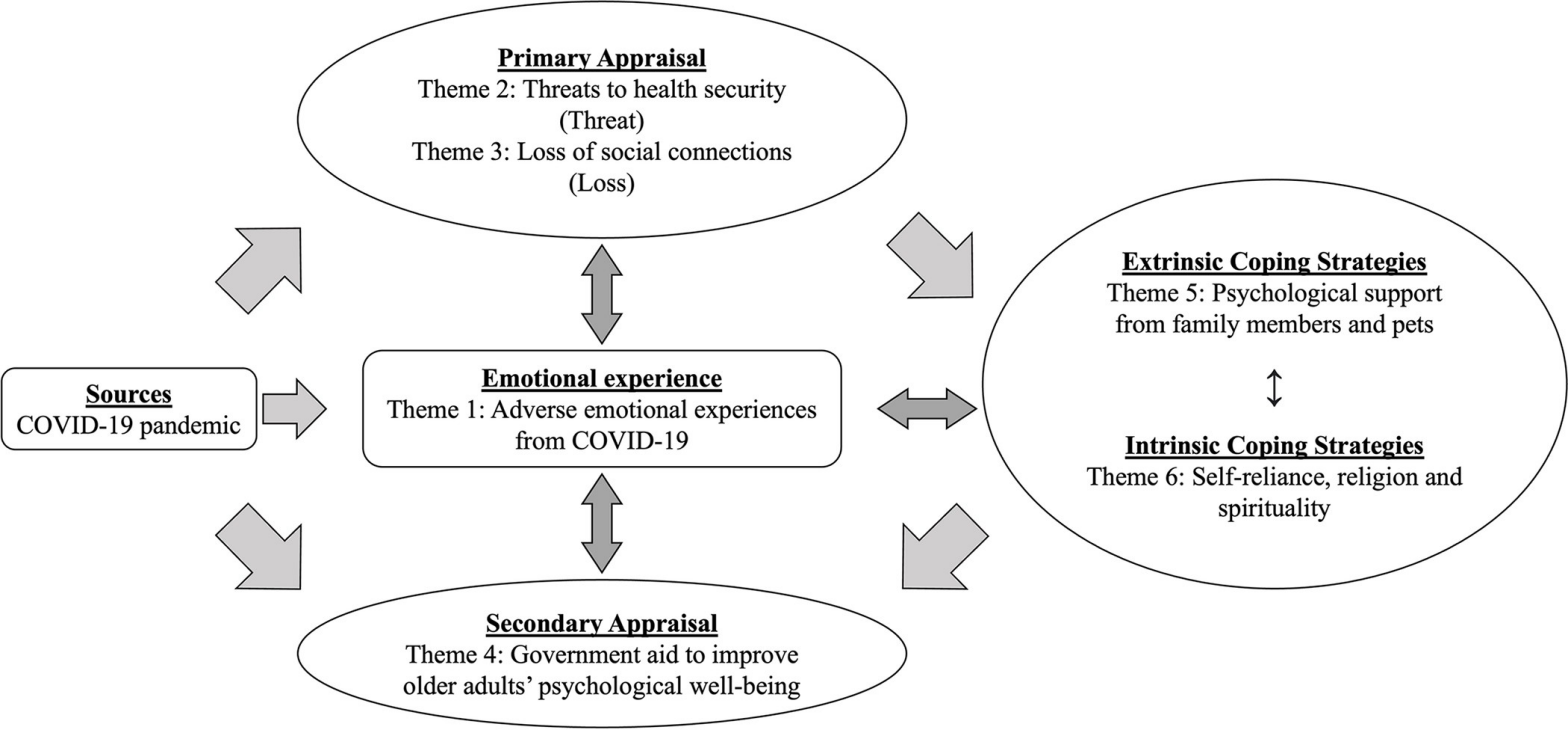
The emotional-transactional model of COVID-19 on vulnerable older adults in Malaysia.

The thematic analysis revealed six themes that were: (1) Adverse emotional experiences from COVID-19, (2) Threats to health security, (3) Loss of social connections, (4) Government aid to improve older adults’ psychological well-being, (5) Psychological support from family members and pets, and (6) Self-reliance, religion, and spirituality. The six themes are discussed in detail below. The themes are organised and presented according to the emotional-transactional model (in Fig 4, the themes are overlaid on the emotional-transactional model).

#### Emotional experience(s)

*Adverse emotional experiences from COVID-19 (Theme 1)*. Most of the participants experienced negative psychological states during the lockdown. Participants expressed negative emotions described as stress, worry or anxiety, fear or being scared, resignation, and wanting to avoid contact with people. As a result of complying with the MCO, respondents commented on how it had negatively impacted both their social and physical state as well.

“*Ya*, *those were the depressing side*. *I have an 84 yr old mother whom I couldn’t visit*, *she has only two daughters—me and my sister—and she wanted to see us*, *we could not and that depressed me a lot*. *These things pulled me down*. *I’m a people’s person*, *not being able to physically touch and see friends and family is what pulled me down emotionally*.*”* (P027)

#### Primary appraisal

*Threats to health security (Theme 2)*. Many of the participants expressed their concerns due to the inability to gain access to health facilities like hospitals and clinics.

“*I think where the Government clinics are concerned*, *they should make it a bit easy because ever since COVID-19 they are making it a bit difficult for people*… *Even if I want to go directly to see the doctors*, *it is not as easy as it used to be before*.*”* (P005)

*Loss of social connections (Theme 3)*. Several participants emphasised the self-isolation and loneliness; there was a sense of resignation to their fate and fear echoed in their quotes.

“*Definitely*, *I am worried… It has affected me psychologically in the sense … As they (the government) kept extending the MCO*, *only then I told myself don’t be a pretender because when you live alone and when depression hits you*, *that’s it*! *It will be very difficult for you to pick up*.*”* (P007)

#### Secondary appraisal

*Government aid to improve older adults’ psychological well-being (Theme 4)*. When asked for suggestions to improve older adults’ psychological well-being, most respondents stated that it was imperative for the Government to step in with suitable interventions and programmes, mainly in the healthcare systems, providing retirement facilities and benefits.

*Healthcare systems*. The majority of participants deemed accessibility to healthcare systems, such as having regular check-ups and visits to the clinic, as important factors to ensure that older adults’ emotional health is supported.

“*… every senior citizens should have medical checkups for free… It’s not cheap*. *So if Government can give full medical yearly*, *would be good*.*”* (P020)

A few suggested the government step up their efforts by building more hospitals and clinics in rural areas.

“*… more hospital in the rural areas or maybe clinics where people can come visit… We want the doctors to be stationed or maybe if we can have 1 or 2 doctors maybe give extra Medical Officers … if the Government really want to take care of the health of the people*, *then the infrastructure of the health must be think of*.*”* (P009)

*Retirement facilities and benefits*. Retirement homes and facilities was one suggestion given by participants to combat self-isolation and reduce depression among older adults.

“*… if we can have a safe space for them and they are happy*, *they have a retired village they can mingle and feel useful*. *They can contribute and they are happy*. *I think the Government should spend chunks and chunks of investment and money on retired homes*.*”* (P015)

The benefits from living in a housing community structure would allow older adults to socialise and encourage them to be involved in physical activities. Many participants highlighted the need for older adults to be engaged with others and not be isolated.

“*Older adults*, *I think the best medicine is go out*, *have lots of friends*, *must socialise to keep your mind active*, *physically active and go out and do physical activities*. *I love gardening and walking*. *Living in a retirement place keeps you in touch with other people so you don’t feel lonely*, *get a lot of ideas*. *… People keep you alive in a healthy way*.*”* (P021)

#### Extrinsic coping strategies

*Psychological support from family members and pets (Theme 5)*. Amongst other requirements during the MCO, the public was restricted to a 10-km radius of their homes and only a single member of each household could purchase essential items (food and medicine) [83]. In general, the participants used either extrinsic or intrinsic coping strategies to overcome their emotional challenges. It has been argued that while intrinsic motivation reflects the human natural propensity to assimilate and learn, extrinsic motivation varies it is relative autonomy, from external control to self-regulation [84].

As a result of these challenges, most of the extrinsic suggestions shared by respondents involved psychological support from family members and having pets. The emotional support provided, either through physical or non-physical connection, helped participants to deal with stress and to combat loneliness.

“*… having your family around*, *my wife and my daughter*. *I guess if you are to face this alone and stay at home alone*. *You probably feel more stressed*, *with no one to talk to and no one there with you*. *I think that helps a lot*.*”* (P028)

Some suggestions of having pets as a companion to cope with the lack of physical contact and connection helped alleviate the stress that comes from being isolated.

“*You stop thinking about yourself and think about others… I have my pets here so when I get up in the morning*, *I am fully occupied with caring for them… Helps maintain my health through the lockdown*.*”* (P005)

“*I relief myself by tending to the garden and pet my cat*. *My husband has birds*. *… when we stand in front of the birds’ cage*, *we see their behaviour*, *their way of life and how they treat other birds*. *Actually*, *birds are loving*. *When we see their behaviour*, *it can make us laugh and entertain us*. *So*, *I don’t feel bored*.*”* (P006)

#### Intrinsic coping strategies

*Self-reliance*, *religion*, *and spirituality (Theme 6)*. Many respondents highlighted their efforts to keep themselves active, both mentally and physically, as a coping mechanism, being self-reliant and turning to religion.

“*… when you keep yourself busy*, *you don’t feel stress all that*. *Your mind is occupied*. *As I get up in the morning up to the time I am sleeping*, *I am fully occupied with the housework*, *and I do exercise too*.*”* (P005)

A number of participants stressed that they did not require psychological support from others to cope and they relied on themselves to overcome any challenges.

“*I turn to myself (for support)*. *That’s the most important thing and I do pretty good because no one can help you if you can’t support yourself*. *That’s what I think*. *I’ve been independent since I was young*.*”* (P022)

Others turned to religion as a coping mechanism during the MCO, claiming that praying or reciting prayers helped them deal with being quarantined.

“*I was praying more and listening to God more*. *I was more into prayers and reading the bible*. *I felt like God was prompting to me ‘Nothing is going to happen*.*’ Just keep praying*. *I felt comforted and no more sadness*.*”* (P013)

## Discussion

Women and older adults in the current study reported experiencing poorer psychological well-being during the pandemic and some of the specific challenges encountered during the MCO. Both groups provided extrinsic and intrinsic measures that either alleviated or prevented negative feelings during MCO and stressed the need for more social protection and fiscal aid to support their well-being. Women specifically reported experiencing a higher incidence of family violence, in addition to financial and economic stress and increased household responsibilities.

These findings are consistent with studies where reduced social interaction from movement restriction measures were associated with a decline in mental well-being, such as increased feelings of loneliness and isolation [2,46,85–91]. The effect of prolonged isolation has been identified as a risk factor for psychological conditions such as major depressive disorders (MDD) and post-traumatic stress disorder (PTSD) [92], further reinforcing the importance of assisting vulnerable groups for whom resources may be particularly inaccessible (both before and during COVID-19).

### Women

Understanding and identifying the impact of COVID-19 using the social ecological framework [70] is useful in the development of strategies to ameliorate the burdens faced by Malaysian women. The inclusion of cross-cutting factors provides insight into women-focused strategies and interventions to reduce the risk of poorer well-being. Importantly, these cross-cutting factors are derived from participants themselves, which makes the recommendations more valuable and potentially more effective.

The results indicated that women perceived a decline in their psychological well-being with the threat of the pandemic and the effects of the MCO. In addition to the emotional challenges brought about by the pandemic, family violence contributed to the decline in mental well-being of women. Participants supported this as they reported increased family tensions from spending more time together, and concern for their well-being and those of their loved ones with their more aggressive family members. The stress of living together with unrelieved contact places individuals at risk, especially at a time of global crisis where there are shortages of essential resources and economic instability [93]. The main reason for family violence in this study was financial stress, which corroborates a Malaysian report citing financial difficulties as one of the main contributing factors for domestic violence [42,94]. Essentially, movement restriction measures inadvertently increased exposure of victims to perpetrators, who face limited consequences for their actions as victims would be unable to seek refuge or access support [94–97].

A deep recession was also predicted in the aftermath of the COVID-19 pandemic, and this economic uncertainty has led to mass layoffs globally [98,99]. In Malaysia, men’s employment was reported to be recovering as movement restrictions were gradually lifted, but the number of employed women continued to decrease [100]. The lack of labour force participation is linked to the increased burden of unpaid care work, which includes household chores and childcare [36]. This inequality is exacerbated by traditional gender roles in Malaysia, which sees women assuming more caregiving responsibilities than men, and having lower-income jobs that were of lower priority and more sensitive to the impact of COVID-19 [35,101,102]. Women would then be more vulnerable to financial and economic insecurity, which subsequently affects their psychological well-being [103] as they will be rendered dependent on others.

Self-coping strategies employed by women included leisure activities and hobbies, along with religious or spiritual practices (e.g., meditation), and reliance on social support. These findings were consistent with a study that suggested Malaysians relied on positive coping strategies to handle the stress of COVID-19 [104]. However, a notable lack of access to mental health resources was identified by participants of the study, and this shortcoming would likely worsen the psychological distress faced by those who are already disadvantaged prior to the pandemic [105].

Progressive empowerment of women to address Malaysia’s gender-based inequalities was suggested by participants and supported by existing research [106]. Empowering women by allowing them to voice their concerns would not only benefit them individually but improve their decision-making and communication skills [107,108]. Women who participated in the current study also believed that their empowerment would be improved by more reasonable actions taken by the government to tackle these inequalities through corporate, fiscal, and social protection, rather than providing degrading and embarrassing domestic advice.

### Older adults

Predictably, movement restriction measures proved detrimental to psychological well-being, and there was an increased need for psychological support globally [1]. For the older adult in this study, limited social engagement affected not only themselves but family members and resulted in feelings of depression with each MCO extension. This is unsurprising, as research has identified similar instances of psychological distress amongst older adults who have been socially isolated due to COVID-19 [109,110]. The lack of trust in technology [111] and poor connectivity [112] further isolated older adults, and this is augmented when considering the collectivistic Malaysian family dynamic [113], where older adults are often cared for by younger family members and the community.

To cope, older participants expressed intrinsic and extrinsic coping strategies that motivated them to overcome emotional challenges brought about by COVID-19. Extrinsically, participants mostly relied on psychological support from family members and companionship from pets, which eased loneliness and stress. The activities reported by participants support the findings of previous studies regarding the benefits of human and non-human companionship during COVID-19 [13,114–116]. Intrinsic coping strategies utilized by participants in this study included maintaining some form of mental and physical activity or a dependency on themselves or religion. This indicates that participants did not necessarily see movement restriction measures as inherently negative but perceived it as the opportunity to make time for spiritual activities and self-reflection [115]. Research indicates that older adults who engaged in intrinsic religiosity tend to experience better physical and mental health than older adults who are not as religious [117–119]. Religious participation has been emphasized as one of the forms of social support the older adults gain in the way of perceived emotional support, which plays a critical role in the maintenance of mental health [120].

Coping strategies for older adults in this study emphasized accessible, subsidized healthcare to maintain physical health. As Malaysia has recently become classified as an ageing society, this requires immediate and extensive planning to ensure that the nation is adequately financially and socially prepared for the implications of this demographic shift [121]. Aside from maintaining physical health, older adult participants voiced the potential for government aid to establish retirement facilities to combat social isolation. The benefits of such retirement facilities have been well-documented in maintaining cognitive function, reducing psychological distress, and improving overall well-being [122–125].

### Thematic links between women and older adults

To summarize, the themes identified in this study have indicated some degree of overlap between both groups (see Table 2). There was an indication that both groups experienced some form of psychological distress arising from COVID-19, but women reported more health-related anxiety in terms of spreading COVID-19 whilst older adults were concerned about accessibility to healthcare. There was also an overlap in terms of coping strategies during the MCO as participants from both groups reported intrinsic and extrinsic coping strategies that helped reduce loneliness from prolonged social isolation. Lastly, the two groups agreed that more government aid should be provided, but they diverged in terms of the form of government support expected, with women advocating for fiscal and social aid but older adults requiring more healthcare-based aid.

**Table 2:** Thematic links between women and older adults.

Theme	Women	Older Adults
Challenges	Psychological	Emotional
Threats	Family violence	Health security
	Financial and employment stress and anxiety	Social connections
	Institutional inequality & prejudice	
Coping strategies	Intrinsic coping strategies	
	Extrinsic coping strategies	
Governmental support	Professional support	Healthcare system reforms
	Financial support	Retirement facilities and benefits

### Implication for theory and practice

At least three practical implications can be suggested based on the findings of the current study. The first concerns the nation, followed by one each for women and older adults. Due to COVID-19 safety guidelines, these recommendations can be adapted online to ensure physical distancing. This is, of course, contingent on improvements of infrastructure by the government [126].

Firstly, intervention programs that focus on building resilience through positive psychology exercises can be developed and implemented, such as the PERCE model [4]. By implementing a more holistic approach that is more focused on intrinsic protective factors, individuals would be better equipped to manage distressing situations that may require physical distancing. Moreover, positive psychology interventions have been reported to protect against psychological distress and enhance positive life experiences that would improve overall psychological well-being [127,128]. The government may also want to consider promoting mental health assistance during the COVID-19 pandemic, to enhance help-seeking efficacy and reduce stigmatization of mental health (i.e., psychoeducation) [129].

Secondly, the introduction of a platform for women to express their concerns safely and find support in a community of like-minded women could be developed, to improve economic, social, and political empowerment [107,108]. This platform may adopt a multiple streams framework to improve accessibility to resources in case of domestic violence, and thus protect women against their aggressors [130]. To reduce the financial strain to develop this platform, it can be proposed that several NGOs collaborate so that resources can be shared. The government can then seek to rectify gender-based labour market disparity, through regulatory initiatives and social campaigns [131,132].

Lastly, there is a need for reformation of Malaysia’s present support for older adults, as the current pension and welfare systems would increase the burden of social expenditure in the future [133]. Delivering a mobile health app (mHealth) for healthcare would encourage older adults to maintain some form of active lifestyle [134], and effectively manage their health [135,136]. In addition to preparing institutional care centres and improving access to healthcare for rural-based older adults [121], the government would benefit from pre-emptively encouraging a healthy ageing process. Ensuring that adults age healthily is crucial for maintaining quality of life and allows them to thrive in society (e.g., as grandparents, or friends) [49,137].

### Limitations and recommendations

The limitations of this study should be taken into consideration when interpreting the results. The research sampling (i.e., women and older adults in B40 households), although adequate to explore the research objectives within the context of the study, is not representative of the entire population. Hence, it would not be appropriate to generalise the findings to other samples. Two recommendations may be made from this limitation: 1) studies focusing on women and older adults belonging to middle (i.e., M40) and high (i.e., T20) income households would provide an opportunity to ascertain the similarities and differences in psychological experiences during MCO, 2) follow-up studies on other vulnerable groups in low-income households (e.g., children, migrant workers) would provide a thorough picture of the impact of COVID-19.

The qualitative approach used for this research provides a very nuanced, subjective view. A recommendation would be to provide additional support in the form of quantitative data or perhaps designing longitudinal research so the long-term implications of COVID-19 pandemic on vulnerable groups can be assessed. Lastly, the mitigation strategies identified here are provided from the perspective of participants, and the usefulness of these strategies would require testing and validation from mental health professionals, psychologists and others working within relevant support structures. Hence, a recommendation would be to replicate these interviews with professionals to assess the efficacy and feasibility of these strategies.

## Conclusion

The qualitative findings of this study support the prevailing evaluation that COVID-19 and movement restriction measures had a negative impact on Malaysian women and older adults in low-income households. Despite the effectiveness of movement restriction measures in mitigating some of the impacts of COVID-19, both groups reported consequences for their mental well-being and shared their expectations for the government to provide aid. Women reported specific additional challenges related to family violence, increased household responsibilities, and a lack of support in the labour force. Prolonged isolation can increase risks to individuals while attempting to protect them, so the mitigations proposed by participants require urgent attention by relevant parties.

## Supporting information

S1 Appendix Interview questions. (DOCX)
