# Supplementary material for: Perceptions of risk and coping strategies during the COVID-19 pandemic among women and older adults
Source: PLoS One. 2024 Apr 17;19(4):e0301009. doi: 10.1371/journal.pone.0301009 (PMC11023439; doi:10.1371/journal.pone.0301009)
Supplement: S1 Appendix — (DOCX) [file pone.0301009.s001.docx]

**Appendix S1**

Interview Questions

1. **What is your experience with government measures related to MCO?**
   1. Have you been exposure to the risk of being affected?
   2. Have you ever thought of changing jobs (keeping/resigning) because of COVID-19 outbreak?
   3. Do you feel that you would want to avoid being in contact with families and friends because of your work?
   4. Have you worried about yourself or your family members being infected by COVID-19?

1. **What challenges have you experienced due to pandemic crisis and MCO?****(psychological/emotional/feelings)**
   1. How would you define psychological challenges in your own word?
   2. What would these emotional challenges mean to you?
   3. How do you deal with these psychological challenges?
2. **What kind of psychological support will be most useful?**
   1. Are you aware of any support that you can access? (psychological/ emotional/ feelings)
   2. Can you name some support system?
   3. What types of support will help you to deal with emotional challenges?
   4. Have you accessed/ experienced to any psychological support?
   5. What type of support have you benefited from the past?
3. **What kind of training or personal development would be beneficial at this particular time? (Training/ development workshop)**
   1. Think about your work and personal life
   2. Can you describe your personal and work related goals?
   3. What type of training or workshop will be useful?
   4. What kind of training you need to improve your quality of life?
   5. What past support of training you already received from your environment (job or communities)?
   6. What kind of additional support that you will find useful?

1. **Do you have any hopeful suggestions that would improve your psychological well-being and the quality of life of elderly/ women/ self-employed workforce?**
   1. If so, please describe what are the suggestions.
   2. How do you think your suggestion could be helpful in COVID-19 situation?
   3. What do you think is the most important support or issue to address post COVID-19?
